# Representative Public Health Surveys Pose Several Challenges: Lessons Learned Across 9 Communities During the COVID-19 Pandemic

**DOI:** 10.1016/j.focus.2024.100198

**Published:** 2024-01-30

**Authors:** Jeanne W. Lawless, Diego G. Diel, Bettina Wagner, Kevin J. Cummings, Genevive R. Meredith, Lara Parrilla, Elizabeth F. Plocharczyk, Robert Lawlis, Samantha Hillson, Benjamin D. Dalziel, Jeffrey W. Bethel, Jane Lubchenco, Katherine R. McLaughlin, Roy Haggerty, Kathryn A. Higley, F. Javier Nieto, Tyler S. Radniecki, Christine Kelly, Justin L. Sanders, Casey L. Cazer

**Affiliations:** 1 Department of Public and Ecosystem Health, College of Veterinary Medicine, Cornell University, Ithaca, New York; 2 Department of Population Medicine and Diagnostic Sciences, College of Veterinary Medicine, Cornell University, Ithaca, New York; 3 Cayuga Medical Center, Ithaca, New York; 4 Cayuga Health Partners, Ithaca, New York; 5 Tompkins County Health Department, Ithaca, New York; 6 Department of Integrative Biology, College of Science, Oregon State University, Corvallis, Oregon; 7 Department of Mathematics, College of Science, Oregon State University, Corvallis, Oregon; 8 College of Public Health and Human Sciences, Oregon State University, Corvallis, Oregon; 9 Department of Statistics, College of Science, Oregon State University, Corvallis, Oregon; 10 College of Science, Oregon State University, Corvallis, Oregon; 11 Center for Quantitative Life Sciences, Oregon State University, Corvallis, Oregon; 12 College of Engineering, Oregon State University, Corvallis, Oregon; 13 Carlson College of Veterinary Medicine, Oregon State University, Corvallis, Oregon; 14 Department of Geology and Geophysics, Louisiana State University, Baton Rouge, Louisiana

**Keywords:** Public health surveillance, cluster sampling, university-community partnership, COVID-19, SARS-CoV-2, serological surveillance

## Abstract

Community surveillance surveys offer an opportunity to obtain important and timely public health information that may help local municipalities guide their response to public health threats. The objective of this paper is to present approaches, challenges, and solutions from SARS-CoV-2 surveillance surveys conducted in different settings by 2 research teams. For rapid assessment of a representative sample, a 2-stage cluster sampling design was developed by an interdisciplinary team of researchers at Oregon State University between April 2020 and June 2021 across 6 Oregon communities. In 2022, these methods were adapted for New York communities by a team of veterinary, medical, and public health practitioners. Partnerships were established with local medical facilities, health departments, COVID-19 testing sites, and health and public safety staff. Field staff were trained using online modules, field manuals describing survey methods and safety protocols, and in-person meetings with hands-on practice. Private and secure data integration systems and public awareness campaigns were implemented. Pilot surveys and field previews revealed challenges in survey processes that could be addressed before surveys proceeded. Strong leadership, robust trainings, and university–community partnerships proved critical to successful outcomes. Cultivating mutual trust and cooperation among stakeholders is essential to prepare for the next pandemic.

## Highlights

• Surveillance surveys can provide timely information on public health threats.
• Developing strong partnerships is essential for successful surveillance.
• Public-health trained staff can address the COVID-19 pandemic and future pandemics.

## INTRODUCTION

Early during the coronavirus disease 2019 (COVID-19) pandemic, public health practitioners primarily relied on test positivity and case counts to monitor the spread of severe acute respiratory syndrome coronavirus 2 (SARS-CoV-2).^1^ However, these approximations of disease incidence and prevalence are biased by unequal test availability and access as well as asymptomatic infections.^2^ Surveillance surveys with representative sampling methods have revealed much higher and more reliable estimates of COVID-19 infection.^1^^,^^3^ Additionally, assessing samples for SARS-CoV-2–specific antibodies improves reliability of population prevalence estimates of natural infection.^1^ The main objective of these surveillance surveys was to provide timely, reliable prevalence estimates to local public health officials to inform their decision-making efforts in response to the COVID-19 pandemic. Another objective was to develop a scalable system that could be rapidly deployed in other communities. This paper highlights lessons learned that may prove beneficial to future surveillance teams.

## METHODS

**Figure 1:**
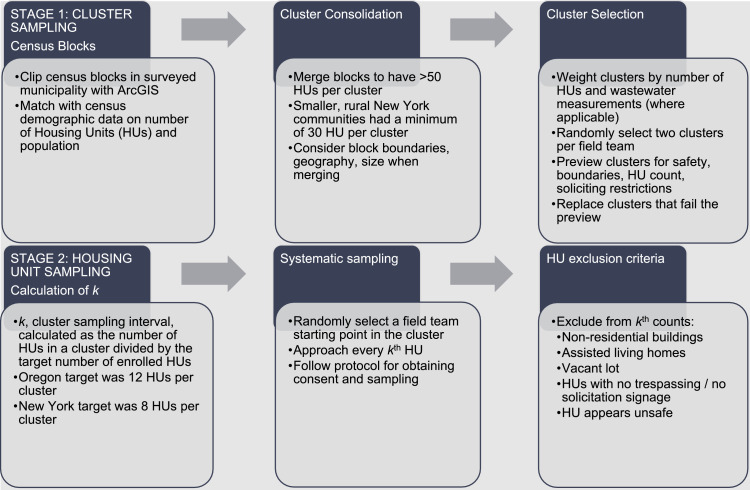
Two-stage cluster sampling design process, OR (2020-2021) and NY (2022).

For rapid health assessment using a representative sample, Oregon State University researchers developed a 2-stage cluster sampling design^4^; it was adapted for use in New York (NY) by another team at Cornell University (CU) (Figure 1). Census tracts were combined into clusters of >50 housing units (HUs) (Oregon [OR]) or 30 HUs (NY) per community surveyed.^5^ Clusters were weighted by the number of HUs, and a sample of clusters was randomly selected. In 3 OR communities, neighborhood-scale wastewater testing that quantified SARS-CoV-2 concentrations was used to adjust the sampling weights to target suspected hotspots.^6^ The second stage of the cluster sampling design took place in the field. A sampling interval, *k*, was calculated by dividing the number of HUs in the cluster by the cluster enrollment target (12 HUs in OR and 8 HUs in NY). Field teams systematically sampled every *k*th HU in each cluster.

Between April 2020 and June 2021, a total of 4 communities in OR were surveyed once, 1 was surveyed twice, and 1 was surveyed 7 times. In NY, 3 communities were each surveyed at 1 time point (February, April, or October 2022). Individuals aged >2 years residing in qualifying HUs were allowed to participate with appropriate consent. HU participation rates in OR communities ranged from 38% to 80%, whereas those in NY ranged from 39% to 46%. Overall, the OR team sampled 7,075 individuals residing in 4,167 HUs. In NY, 233 individuals residing in 221 HUs were sampled.

Consenting individuals provided self-collected nasal swab samples. Samples were tested for SARS-CoV-2 using EZ-SARS-CoV-2 real-time RT-PCR^7^ (NY) and ThermoFisher Scientific TaqPath COVID-19 diagnostic kit (OR) and for nucleocapsid and receptor binding domain antibodies by multiplex assay^8^ (NY and 1 OR survey). Participants provided demographic information, symptom history, and vaccination status. NY surveys included questions on SARS-CoV-2 prevention behaviors and attitudes. A more detailed descriptions of methods (NY) is available.^9^

The CU (#2107010440) and the Cayuga Medical Center IRBs (#0821EP) reviewed the NY arm of the study and determined it to be not human participant research under 45 CFR 46.102(1)(2). The Oregon State University IRB approved the surveys in OR, initially as human subjects research (#2020-0633) and then as public health surveillance activities under 45 CFR 46.102(1)(2) (#2020-0772).

## RESULTS

Considerable preparatory efforts were invested to ensure safe, successful surveys consistent with community public health best practices and the needs of local public health officials. Lessons learned are summarized in Table 1. Each of the strategies adopted were instrumental to the success of the survey, the protection of the participants, and the safety of the staff. Survey leaders determined which lessons learned and methods would be most useful to future surveys and other surveillance teams through an iterative discussion and feedback from the field staff.

**Table 1:** Methodologic Challenges, Resolutions, and Specific Examples, OR (2020–2021) and NY (2022)

Challenge	Resolution	Specific examples
Field staff training and safety	■ Comprehensive field manuals (NY:^9^) ■ Combination of asynchronous online, synchronous hybrid (in-person and Zoom), written, and fully in-person training modalities ■ Protocol development input from Environmental Health and Safety ■ Safety and communication apps ■ Field staff empowered to leave unsafe areas ■ Reflection sessions with field staff to improve safety	■ IRB, HIPAA basic training ■ Public health communication ■ LARA Technique: Listen, Affirm, Respond, Add ■ PPE (Personal Protective Equipment) Use ■ Sample collection, spills, and transport protocols ■ Sample spill kit protocol ■ Emergency contact list ■ Personal safety alarms ■ Live location pins on mobile phone app ■ Field staff debrief reflections: ○ Politely leaving properties/neighborhoods ○ Practice communicating with frustrated property owners and people ideologically opposed to COVID-19 interventions
Consistent field survey methods	■ Field manual of survey methods ■ Pilot survey to test methods ■ Location previews to assess accessibility and mobile data reception ■ Senior staff check-ins with teams in the field ■ Reflection sessions with field staff to improve methods	■ Cluster maps ■ Housing Unit tracking sheets ■ Supply checklists ■ Sample tracking system using bar codes, color-coding and tracking sheets ■ Tablet with hotspot for data collection ■ Backup system with paper and/or offline tablet app ■ Field staff debrief reflections: ○ Field staff preview clusters in advance to locate HUs ○ Pilot methods on community members to troubleshoot the types of questions asked ○ Shorter survey and avoiding sensitive questions ○ Pair experienced and new field staff to improve confidence and performance ○ Organized supplies, checklists, and printed maps are helpful ○ Survey availability in multiple formats (tablet vs paper vs read aloud) encourages participation
Private/secure data integration systems	■ Secure digital data collection ■ Integration with existing HIPAA-compliant system (NY) ■ Protect subjects using separate personal identifiers for survey data and test results	■ Use of passcode-protected tablets ■ Need for sophisticated technical support linking medical and university data ■ Community partners coordinator
Public awareness promotion	■ Website ■ Survey branding ■ Communication briefings with ○ Campus police ○ Community police ○ Public safety personnel ○ Municipal officials ○ Local health departments	■ Press release to community ■ Field staff ○ T-shirts with survey branding ○ Name tags with university/survey affiliation ■ Leave-behind door hangs and information sheets ○ Provide contact information for survey leadership ■ Health department informational flyers ■ News media handouts ■ Need for canvasing/soliciting permits in some locations
Inclement weather	■ Preparing team and vehicles for expected weather conditions ■ Previewing locations for unsafe roads ■ Team adjustments due to inclement weather absenteeism	■ Winter storm preparation ○ Hybrid online and in-person delivery of training ○ Extra vehicles ○ Snow shovels, windshield scrapers, and hand warmers ○ Water and breaks during hot weather ■ Ponchos and umbrellas for rainy weather
Diverse communities and partners	■ Early discussions with local public health officials ■ Engagement of extension faculty to learn about local community ■ Multimedia advertising of the project in advance of data collection ■ Hire local and bicultural/bilingual field staff	■ Community Partners Coordinator ○ Facilitates relationship between university and community partners ■ Inclusion of local health department questions in survey development ○ Addresses unique concerns of the community

*Note*: HIPAA, Health Insurance Portability and Accountability Act of 1996; HU, housing unit; NY, New York; OR, Oregon.

### Field Staff Training and Safety

Field staff included graduate and undergraduate students and community members. Trainings included ethical conduct in human subject research, consent procedures, Health Insurance Portability and Accountability Act of 1996, personal protective equipment, sample handling and transport, and waste disposal procedures (Table 1). Effective communication strategies were presented and practiced, including the listen, affirm, respond, ask (LARA) method. Safety precautions, including de-escalation training, personal awareness, safety alarms, and location monitoring, were reviewed in written materials, presentations by campus safety officers, and in-person trainings. The importance of communication training and practice was emphasized in feedback from field staff on their experiences conducting the surveys (Table 1).

### Field Survey Method Consistency

Survey administration consistency was achieved through comprehensive field manuals and team leader continuity. Methods and survey instruments were piloted and refined to ensure they were logistically feasible, appropriate for the community, and in compliance with all regulations. Clusters were previewed by survey leaders to evaluate safety, access, mobile internet availability, and prohibitions on solicitations and replaced where necessary (Table 1). Debriefings with field staff after each survey and at the end of the project helped to identify areas for improvement and clarification (Table 1).

### Private/Secure Data Integration Systems

Password-protected tablets were used to securely collect Health Insurance Portability and Accountability Act of 1996–protected (NY) and/or personally identifiable health information. Secure tablets may not be available immediately in a public health emergency. The first 10 surveys conducted in OR used paper forms. Trained personnel entered information into a secure database. A separate survey on infection history, demographics, and behaviors was administered in NY. Laboratory test results were linked to survey data using a de-identified sample number (OR and NY; Table 1).

### Public Awareness Promotion

The public was made aware of surveys through media releases and websites (Table 1), which were posted in English (OR and NY) and other languages spoken in the surveyed community (OR). Media were given access to team leaders but were not told sampling locations to maintain participant privacy. A flyer with survey information and revisit date/time was left at selected HUs if no adult was home at the initial visit. Local police and municipal officials were given advance notice of survey dates. Field staff wore name tags and t-shirts, identifying them as survey staff. Aggregate results of infection and immunity prevalence were posted online.

### Inclement Weather

Weather patterns pose unique challenges to surveillance field implementation (Table 1). Considerable forethought must be put into the planning for rainy, snowy, icy, and/or hot weather to ensure the safety of field staff, adequate operation of data systems, and proper handling and storage of samples. Flexibility in research design and execution enables collection of high-quality data despite such challenges.

### Diverse Communities and Partners

To ensure community members were approached respectfully, steps were taken in advance to become familiar with each community. Knowledgeable colleagues at local health departments, police departments, and others contributed to the design and execution of the surveys. Cultural competencies were discussed in field staff trainings, and, in OR, multilingual field staff were hired for all community surveys (Table 1).

## DISCUSSION

Surveillance surveys are cost- and resource-intensive but can provide critical real-time public health data. Based on experiences implementing surveillance surveys, 3 high-priority areas were identified as instrumental to the success of these surveillance surveys: building on existing systems, developing training methods for consistency, and developing partnerships. First, using existing systems such as technology, protocols, and partnerships can ease survey initiation and implementation. For example, existing safety protocols and trainings for university personnel working with SARS-CoV-2 samples were available to use in the surveys (Table 1). In NY, an existing secure data collection system from a community partner was used. The leveraging of existing infrastructures, technology, and students was recently reported as helpful in the pandemic response of 1 university.^10^ Following the West African Ebola outbreak in 2014–2016, building up information technology and digital health resources helped strengthen surveillance and outbreak response and have been called upon to scale up to curb the spread of COVID-19.^11^

Second, consistent methods and availability of trained staff are necessary to ensure safe, high-quality data collection and results. Standard Operating Procedures^12^ and protocols were created to ensure consistency of field methods (Table 1^9^ ) and can be adapted to new communities. The effective use of technology ensured field team safety and survey consistency. Having a public health-trained work force available for surveillance surveys will help address future pandemics. For example, NY State and CU developed Public Health Corps and online public health training for the public.^13^ CU also trained staff and students to conduct SARS-CoV-2 case investigations and contact tracing.^14^ Rigorous student trainings have been reported in another university's pandemic response initiative.^10^ In Morocco, the presence of well-trained rapid response teams have been credited as 1 of 9 major strengths that enabled the country to control SARS-CoV-2 spread early in the pandemic.^15^ Training programs and modules developed during the SARS-CoV-2 pandemic can be modified and scaled for other public health emergencies.

Third, the partnerships developed between universities, communities, public health agencies, and public safety agencies were essential to survey success. The support of university leadership was critical to prioritizing rapid response actions and quick risk-management decisions. Building upon existing university-community partnerships and forging new ones were possible in these settings because of mutual trust and cooperation developed through years of prior positive interactions. Partnerships have been reported as critical to pandemic response successes as seen in laboratory operations,^16^ between public health agencies and traditional healthcare facilities,^17^ university operations,^18^ and with members of the community.^11^^,^^15^^,^^17^^,^^19^ Community partnerships established through public awareness endeavors result in inclusion of members of the public; robust community engagement cannot be overlooked in surveillance endeavors.^11^^,^^15^^,^^17^^,^^19^

These findings are limited by the small number of communities surveyed in 2 states; other communities and locations may encounter different challenges.

## CONCLUSIONS

Representative surveillance surveys enable more comprehensive case detection and knowledge of disease prevalence to further mitigate spread of disease. By providing timely prevalence information, surveillance surveys continue to serve as a cornerstone of public health practice^20^ to significantly improve local and regional public health responses. Engaging public stakeholders in collaborative ways that cultivate partnerships as sustainable commodities will help prepare for the next public health threat and further the process of putting the public back into public health.

## CRediT authorship contribution statement

**Jeanne W. Lawless:** Data curation, Investigation, Project administration, Visualization, Writing – original draft, Writing – review & editing. **Diego G. Diel:** Conceptualization, Data curation, Investigation, Methodology, Resources, Writing – review & editing. **Bettina Wagner:** Conceptualization, Data curation, Investigation, Methodology, Resources, Writing – review & editing. **Kevin J. Cummings:** Conceptualization, Methodology, Writing – review & editing. **Genevive R. Meredith:** Conceptualization, Methodology, Investigation, Writing – review & editing. **Lara Parrilla:** Methodology, Writing – review & editing. **Elizabeth F. Plocharczyk:** Investigation, Resources, Writing – review & editing. **Robert Lawlis:** Methodology, Resources, Writing – review & editing. **Samantha Hillson:** Methodology, Writing – review & editing. **Benjamin D. Dalziel:** Conceptualization, Funding acquisition, Investigation, Methodology, Project administration, Supervision, Resources, Writing – review & editing. **Jeffrey W. Bethel:** Conceptualization, Funding acquisition, Investigation, Methodology, Project administration, Supervision, Resources, Writing – review & editing. **Jane Lubchenco:** Conceptualization, Funding acquisition, Investigation, Methodology, Project administration, Supervision, Resources, Writing – review & editing. **Katherine R. McLaughlin:** Conceptualization, Funding acquisition, Investigation, Methodology, Project administration, Supervision, Resources, Writing – review & editing. **Roy Haggerty:** Conceptualization, Funding acquisition, Investigation, Methodology, Project administration, Supervision, Resources, Writing – review & editing. **Kathryn A. Higley:** Conceptualization, Funding acquisition, Investigation, Methodology, Project administration, Supervision, Resources, Writing – review & editing. **F. Javier Nieto:** Conceptualization, Funding acquisition, Investigation, Methodology, Project administration, Supervision, Resources, Writing – review & editing. **Tyler S. Radniecki:** Conceptualization, Funding acquisition, Investigation, Methodology, Project administration, Supervision, Resources, Writing – review & editing. **Christine Kelly:** Funding acquisition, Investigation, Project administration, Supervision, Resources, Writing – review & editing. **Justin L. Sanders:** Conceptualization, Funding acquisition, Methodology, Project administration, Supervision, Resources, Writing – review & editing. **Casey L. Cazer:** Conceptualization, Data curation, Formal analysis, Funding acquisition, Investigation, Methodology, Project administration, Supervision, Resources, Writing – original draft, Writing – review & editing.
